# Validation of the 5th edition of the World Health Organization and International Consensus Classification guidelines for *TP53*-mutated myeloid neoplasm in an independent international cohort

**DOI:** 10.1038/s41408-025-01290-0

**Published:** 2025-05-07

**Authors:** Mithun Vinod Shah, Kevin Hung, Anmol Baranwal, Gauri Wechalekar, Aref Al-Kali, Carla R. Toop, Patricia Greipp, Monika M. Kutyna, Aasiya Matin, Dariusz Ladon, Antoine Saliba, Dong Chen, Kebede Begna, Anna Brown, Danielle Rud, Mark R. Litzow, William J. Hogan, Peter Bardy, Talha Badar, Sharad Kumar, David T. Yeung, Mrinal M. Patnaik, James M. Foran, Rong He, Naseema Gangat, Mehrdad Hefazi, Hamish S. Scott, Cecilia Y. Arana Yi, Hassan Alkhateeb, Abhishek A. Mangaonkar, Daniel Thomas, Christopher N. Hahn, Attilio Orazi, Daniel A. Arber, Chung Hoow Kok, Ayalew Tefferi, Devendra Hiwase

**Affiliations:** 1 Division of Hematology, Mayo Clinic, Rochester, MN USA; 2 Department of Haematology, Royal Adelaide Hospital, Central Adelaide Local Health Network, Adelaide, SA Australia; 3 Precision Medicine Theme, South Australian Health and Medical Research Institute (SAHMRI), Adelaide, SA Australia; 4 Adelaide Medical School, University of Adelaide, Adelaide, SA Australia; 5 Laboratory Medicine and Pathology, Mayo Clinic, Rochester, MN USA; 6 Genetic and Molecular Pathology, SA Pathology, Adelaide, SA Australia; 7 Division of Hematopathology, Mayo Clinic, Rochester, MN USA; 8 Centre for Cancer Biology, University of South Australia and SA Pathology, Adelaide, SA Australia; 9 Department of Hematology/Oncology, Mayo Clinic, Jacksonville, FL USA; 10 Hematology/Oncology, Mayo Clinic, Scottsdale, AZ USA; 11 Texas Tech University Health Sciences Center, El Paso, TX USA; 12 University of Chicago, Chicago, IL USA

**Keywords:** Acute myeloid leukaemia, Cancer genomics, Myelodysplastic syndrome

## Abstract

The World Health Organization (WHO-5) and International Consensus Classification (ICC) acknowledge the poor prognosis of *TP53*-mutated (*TP53*^mut^) myeloid neoplasm (MN). However, there are substantial differences between the two classifications that may lead to under- or overestimation of the prognostic risk. We retrospectively applied WHO-5 and ICC to 603 MN cases harboring *TP53*^mut^ (variant allele frequency, VAF ≥ 2%). WHO-5 and ICC would not classify 64% and 20% of these cases as *TP53*^mut^ MN, respectively. Moreover, of those classified, 67.5% would be classified discrepantly. Primary drivers of discrepancies included: (i) prognostic importance of *TP53*^mut^ acute myeloid leukemia (AML), (ii) interaction of the blast percentage and allelic status, (iii) 17p.13.1 deletion detected by cytogenetics, (iv) complex karyotype (CK) as multi-hit equivalent, and (v) *TP53*^mut^ VAF threshold, we analyzed survival outcomes of each of these groups with an aim to provide clarity. *TP53*^mut^ AML was associated with significantly poor survival compared to *TP53*-wild type *TP53*^wt^ AML, myelodysplasia-related (AML, MR 4.7 *vs*. 18.3 months; *P* < 0.0001), supporting its inclusion within *TP53*^mut^ MN as a distinct subentity. Secondly, the survival of *TP53*^mut^ with blast 10–19% was poor regardless of the allelic status. Thirdly, for cases with a single *TP53*^mut^ with VAF < 50%, 17p13.1 del or CK serve as practical surrogates of biallelic inactivation, obviating the need for an additional copy number analysis. Finally, *TP53*^mut^ AML, MDS multi-hit/multi-hit equivalent with VAF < 10% had significantly poorer survival compared to *TP53*^mut^ MDS VAF < 10% without CK and 17p del, and were comparable to those with VAF ≥ 10% (14.1 *vs*. 48.8 *vs*.7.8 months, *P* < 0.0001). Collectively, these findings address key areas of contention and provide valuable insights that will guide future revisions of the WHO and ICC classifications.

## Introduction

*TP53* mutations (*TP53*^mut^) are observed in 5–13% of de novo myelodysplastic syndrome (MDS) [1–6] and 8–11% of acute myeloid leukemia (AML) cases [7, 8]. These mutations are particularly enriched in myeloid neoplasm (MN) following cytotoxic therapies (30–35%) [9, 10], and older patients with MDS and AML (20–40%) [11, 12], being associated with extremely poor survival with limited therapeutic options in contemporary clinical practice [7, 13, 14]. Recognizing the poor prognosis, the World Health Organization (WHO) classifications (5th edition, hereafter referred to as WHO-5) [15] and the International Consensus Classification (ICC) [16] recognized *TP53*^mut^ MDS and MN as distinct entities, respectively.

Both classification systems define an MDS biallelic *TP53* alteration as a distinct subgroup. However, there are notable differences between the two classifications (Table 1). For example, the ICC recognizes *TP53*^mut^ AML as a distinct entity, whereas WHO-5 does not. Both classifications acknowledge the poor prognosis associated with MDS with *TP53* biallelic inactivation; however, they differ in their emphasis on blast percentage. The ICC prioritizes allelic status and blast percentage in risk stratification, while WHO-5 defines biallelic *TP53* inactivation as a homogenous category with poor survival regardless of the blast percentage (0–19%). The ICC requires a VAF of ≥10% for *TP53*^mut^, whereas the WHO-5 does not specify a VAF threshold. In cases with a single *TP53*^mut^ with VAF < 50%, complex karyotype (CK) is considered as multi-hit equivalent by ICC but not WHO-5.

**Table 1:** Areas of discrepancy between the 5th edition of the World Health Organization (WHO-5) and the International Consensus Classifications (ICC).

No.	Area of discrepancy	WHO-5 (MDS with biallelic *TP53* inactivation)	ICC (Myeloid neoplasms with mutated *TP53)*	Evidence presented	Conclusion
1	Acute myeloid leukemia (AML)	No separate category, most cases would be classified as AML-MR	Separate category (BM/PB blasts ≥20%), includes AEL	Survival of *TP53*^mut^ AML is significantly poorer compared to *TP53*^wt^ AML-MR (4.7 *vs*. 18.3 months; *P* < 0.0001).	*TP53*^mut^ AML should be included in *TP53*^mut^ MN
2	Interaction of blast percentage category with allelic status	Requires demonstration of biallelic loss for 0–19% blasts	Requires demonstration of biallelic loss for 0–9% blasts	Survival of monoallelic *TP53*^mut^ MDS with 10–19% blasts is comparable to biallelic loss.	Monoallelic *TP53*^mut^ MDS with 10–19% blasts should be considered *TP53*^mut^ MN
3	Confirmation of 17p13.1 deletion detected on karyotype by CNV analysis	Requires confirmation of loss of the *TP53* locus detected on the karyotype with an additional CNA method	Does not require confirmation of 17p13.1 deletion	**Cases with single** ***TP53***^**mut**^ **(VAF** < **50%) with 17p13.1 deletion on karyotype:** CNV analysis verified the LOH across the *TP53* locus in 94% of evaluable cases. **Cases with single** ***TP53***^**mut**^ **(VAF** < **50%)** **without** **17p13.1 deletion on karyotype:** • CNV analysis identified LOH/cnLOH in 26.9% of evaluable cases. • Importantly, all cases with LOH/cnLOH across the *TP53* locus on CNV analysis had CK. • Conversely, no LOH/cnLOH was observed in cases with or without CK	In a single *TP53*^mut^ with VAF < 50%, confirmation of 17p deletion with an additional CNV is not necessary
4	CK as a multi-hit equivalent	CK is not considered a multi-hit equivalent	CK is considered a multi-hit equivalent	Survival of single *TP53*^mut^ VAF < 50% with CK was comparable to those with 17p deletion on karyotype (10.4 *vs*. 11.0 months; *P* = 0.39) and poorer than monoallelic *TP53*^mut^ without 17p loss or CK (33.4 months; *P* < 0.0001)	In single *TP53*^mut^ with VAF < 50%, CK should be considered multi-hit equivalent
5	VAF threshold	None	VAF ≥ 10%	Survival of *TP53*^mut^ ‘multi-hit’ VAF 2 to <10% was shorter compared to that of ‘single-hit’ *TP53*^mut^ VAF < 10%, but comparable to VAF ≥ 10% (14.1 *vs*. 48.8 *vs*. 7.8 months, *P* < 0.0001).	‘Multi-hit’ *TP53*^mut^ with VAF 2 to <10% should be included in *TP53*^mut^ MN

*AEL* acute erythroid leukemia, *AML* acute myeloid leukemia, *AML-MR* AML with myelodysplasia-related changes, *BM* bone marrow, *CK* complex karyotype, *CNA* copy number analysis, *ICC* International Consensus Classification, *MDS* myelodysplastic syndrome, *MN* myeloid neoplasm, *PB* peripheral blast, *t-MN* therapy-related myeloid neoplasm, *TP53*^mut^ TP53 mutated, *TP53*^mut^ TP53 mutated, *WHO-5* 5th edition of the WHO classification, *VAF* variant allele frequency.

As both classifications are increasingly being used to govern clinical practice, the differences between the two classifications can lead to either under- or overestimation of the prognostic risk, which may translate into inconsistencies in treatment decisions. The lack of consensus likely stems from limited studies that integrate morphological, allelic status, blood and bone marrow blast percentage and genetic features.

In this study, we validated both classifications in our large international cohort with well-annotated clinical data and analyzed how the WHO-5 and ICC criteria impact classification and clinical practice. We also evaluated the factors contributing to the differences between these two classification systems. Finally, we provide evidence that can be used for the revision of both classifications and/or developing a consensus uniform classification system.

## Methods

We analyzed *TP53*^mut^ (VAF ≥ 2%, *n* = 603) MDS and AML patients managed at the Mayo Clinic (USA) and South Australia Health Network (Australia) between February 2002 and August 2024. Data were obtained with informed consent or appropriate consent waiver, in accordance with the Declaration of Helsinki and approved by the relevant Ethics Committee (HREC/15/RAH/496 for South Australia Health Network and IRB# 19-007595/19-007568 for Mayo Clinic).

*TP53*^mut^ was identified by next-generation sequencing (NGS) panels that covered at least exons 4–11. *TP53*^mut^ MDS and AML were retrospectively classified using the ICC [16] and WHO-5 [15] classifications (please refer to supplementary section for details), and their outcome was compared with *TP53* wild type *(TP53*^wt^*)* MN (*n* = 600).

In the WHO-5, MDS with biallelic *TP53* loss is defined by the presence of [1] two *TP53*^mut^; [2] the presence of a single *TP53*^mut^ VAF < 50% with a 17p loss or copy-neutral loss of heterozygosity (cnLOH) verified by CNV analysis. One *TP53* mutation with VAF ≥ 50%, without 17p loss or cnLOH, is considered presumptive evidence of biallelic *TP53* inactivation. Conversely, a single *TP53*^mut^ with VAF < 50% without 17p loss or cnLOH is defined as monoallelic *TP53* inactivation. WHO-5 mandates the confirmation of 17p loss detected on metaphase karyotype by copy number variation (CNV) analysis, such as fluorescence in situ hybridization (FISH) and/or array techniques (single-nucleotide polymorphism arrays) or NGS.

According to the ICC, multi-hit *TP53* inactivation for MDS is defined as [1] two *TP53*^mut^ (each with VAF ≥ 10%), or [2] single *TP53*^mut^ (VAF $\ge$10%) in combination with either (i) deletion of 17p13.1 causing loss of heterozygosity (LOH), (ii) confirmed copy neutral LOH (cnLOH), (iii) presumed cnLOH based on mutation VAF ≥ 50%, or (iv) a complex karyotype. Single hit status is defined as the presence of a deleterious *TP53*^mut^ not meeting the multi-hit criteria. The ICC mandates multi-hit *TP53* inactivation for MDS 0–9% blasts for inclusion as a *TP53*^mut^ MN, while *TP53*^mut^ MDS/AML (10–19% blasts) or AML (≥20% blasts) are included regardless of the hit-status.

### Statistical methods

Comparisons were performed using the Mann–Whitney *U*-test for non-normally distributed variables. Fisher’s exact test was used to determine associations between categorical variables. Wilcoxon rank-sum test or Student’s *t* test was used to compare continuous variables. All statistical tests were two-sided. Overall survival (OS) was calculated from the date of MN diagnosis to the last follow-up or the date of death. Patients alive at the last follow-up date were censored. Kaplan–Meier estimations were used with comparisons using log-rank tests. *P* values < 0.05 were considered statistically significant. All statistical analyses were conducted using the GraphPad and R statistical platform (https://www.r-project.org/) v.4.1.1.

## Results

### Study population and characteristics

The majority of the *TP53*^mut^ MN (*n* = 603) were MDS (*n* = 374, 62.0%) followed by AML (*n* = 229, 38.0%). The median age at diagnosis was 69.0 years (interquartile range, IQR 62.0, 75.0) and 63.4% (*n* = 382) were male (Table 2).

**Table 2:** Clinical characteristics of *TP53*^mut^ MDS and AML cohort.

Variables	Overall (*n* = 603)
Female/male	221 (36.7%)/382 (63.3%)
Age at MN diagnosis, median [IQR]	68.60 [62.00, 75.00]
*Blood counts and marrow blast, median [IQR]*	
Hemoglobin, g/L	9.00 [7.90, 10.50]
WBC	3.00 [1.80, 5.10]
Platelets	55.00 [30.00, 106.00]
PB blasts %	1.00 [0.00, 7.00]
BM blasts %	8.90 [2.50, 27.25]
*Disease phenotype (%)*	
AML	229 (38%)
MDS	374 (62%)
*Cytogenetic changes, n (%)*^*a*^	
Complex karyotype	484 (80.7%)
Monosomal karyotype	437 (72.8%)
Deletion 5q	441 (73.9%)
Deletion 7q	306 (51.3%)
Deletion 17p	240 (40.2%)
*TP53 mutation characteristics*	
*TP53*^mut^ VAF (median, IQR)	36.80% (19.6, 59.0)
*TP53*^mut^ VAF ≥ 2%	603 (100%)
*TP53*^mut^ VAF ≥ 10%	520 (86.2%)
*TP53*^mut^ VAF < 10%	83 (13.8%)
*Somatic co-mutations on NGS, n (%)*	
*TET2*	66 (10.9%)
*DNMT3A*	63 (10.4%)
*ASXL1*	48 (8.0%)
*SF3B1*	33 (5.5%)
*RUNX1*	29 (4.8%)
*SRSF2*	29 (4.8%)
*JAK2*	27 (4.5%)
*U2AF1*	25 (4.1%)
*RAS*	20 (3.3%)
*EZH2*	16 (2.7%)
*BCOR*	15 (2.5%)
*IDH2*	12 (2.0%)
*PTPN11*	11 (1.8%)
*CBL*	7 (1.2%)
*CEBPA*^b^	7 (1.2%)
*ZRSR2*	6 (1.0%)
*First line therapy n (%)*^c^	
Hypomethylating agents	214 (36.4%)
Venetoclax-based therapies	111 (18.9%)
Allogeneic stem cell transplantation	99 (16.4%)
Intensive chemotherapy	96 (16.3%)
Lenalidomide	20 (3.4%)
Erythropoietin/Erythroid differentiation agents	19 (3.2%)
Other therapies	9 (1.5%)
Supportive therapy	111 (18.9%)

*AML* acute myeloid leukemia, *BM* bone marrow, *MDS* myelodysplastic syndrome, *MN* myeloid neoplasm, *NGS* next generation sequencing, *PB* peripheral blood, *VAF* variant allele frequency, *WBC* white blood cell.

^a^Cytogenetics data was not available in three patients.

^b^*CEBPA* mutations were not screened for in five patients.

^c^First-line treatment data were not available in 15 patients.

Compared to *TP53* wild type (*TP53*^wt^), the median OS of *TP53*^mut^ MN was poor independent of CK and blast categories (Supplementary Fig. 1A). In *TP53*^mut^ cohort, allogeneic stem cell transplant was associated with longer OS compared to patients treated with intensive chemotherapy or hypomethylating agents with or without venetoclax (Supplementary Fig. 1B).

### The majority of MDS and AML harboring *TP53*^mut^ are NOT acknowledged as *TP53*^mut^ MN by the WHO-5 classification

**Fig. 1:**
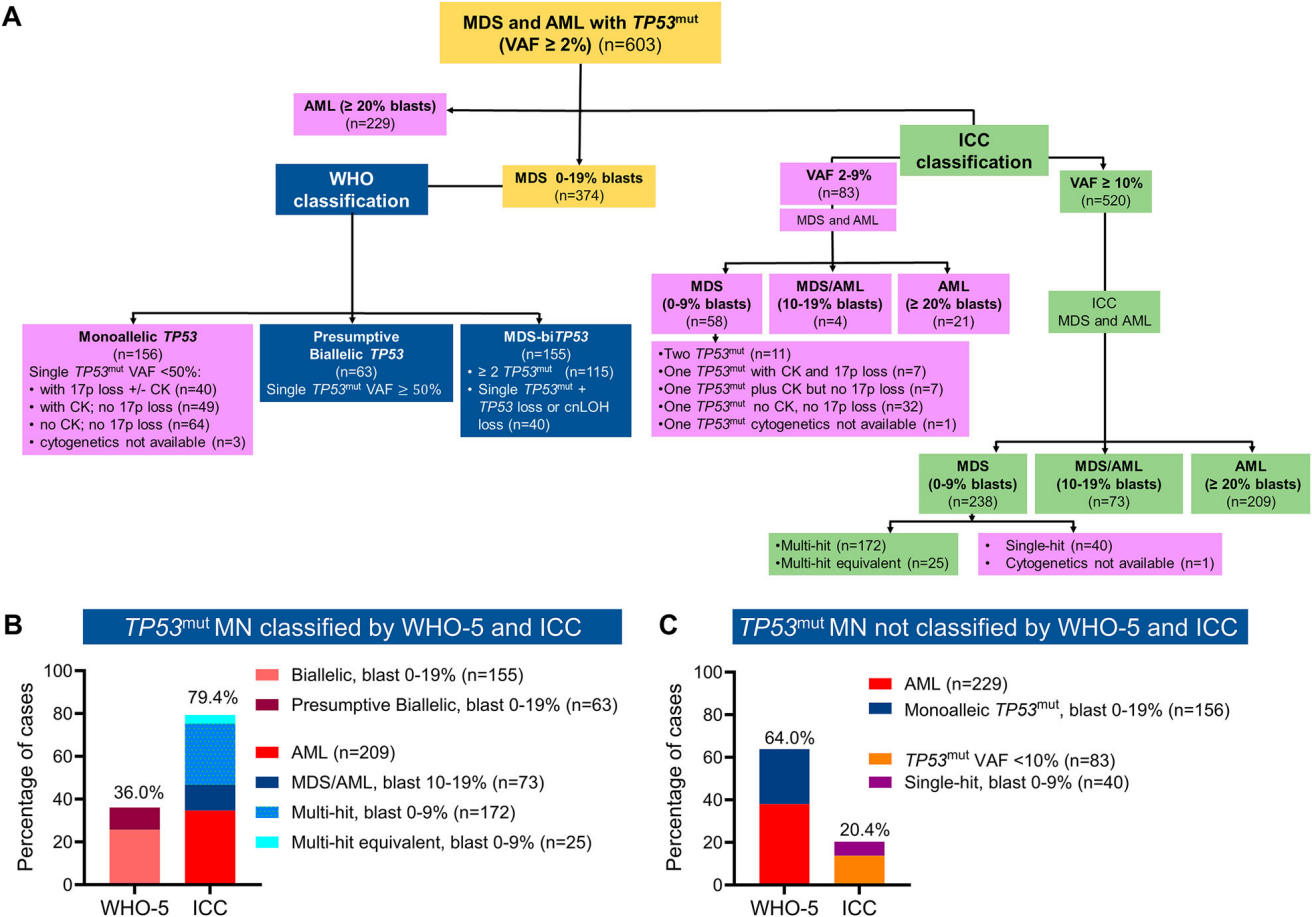
*TP53*-mutated (*TP53*^mut^) myeloid neoplasm (MN) classified by World Health Organization 5^th^ edition (WHO-5) and International Consensus Classification (ICC). **A** Consort diagram summarizing the classification of *TP53*^mut^ MN by WHO-5 (blue) and ICC (green) criteria, along with disease subcategories presently excluded from each classification system, respectively (purple). **B** Percentage of MN with *TP53*^mut^ that can be classified, and **C** not classified using WHO-5 and ICC criteria.

According to WHO-5 criteria, 155 MDS (25.7%) were classified as MDS-bi*TP53* as they harbored $\ge$2 *TP53*^mut^ (*n* = 115) or 1 *TP53*^mut^ plus confirmed LOH or cnLOH of the *TP53* locus (*n* = 40). Additionally, 63 (10.4%) *TP53*^mut^ MDS with VAF $\ge$50% were classified as presumptive MDS-bi*TP53* (Fig. 1A and Supplementary Fig. 2). In MDS with a single *TP53*^mut^ with VAF < 50%, verification of loss of heterozygosity (LOH) or copy-neutral LOH (cnLOH) of the *TP53* locus by CNV analysis is mandatory for defining MDS-bi*TP53* inactivation. In our cohort, 135 (22.4%) cases with single *TP53*^mut^ with VAF $<$50% did not have CNV analysis and thus could not be classified as MDS-bi*TP53*. Moreover, WHO-5 does not classify *TP53*^mut^ AML as a distinct entity. Hence, 229 (38.0%) AML with *TP53*^mut^ were grouped with other AML. In summary, applying WHO-5 would classify only 217 (36%) of 603 as *TP53*^mut^ MN, while 386 (64.0%) were excluded (Fig. 1A–C).

### The majority of MDS and AML harboring *TP53*^mut^ are included in the ICC classification of *TP53*^mut^ myeloid neoplasm

We next performed a similar analysis using the ICC criteria. ICC mandates *TP53*^mut^ VAF $\ge$10% and classifies *TP53*^mut^ MN (*n* = 520) into three subcategories: multi-hit *TP53*^mut^ MDS with BM and blood blast 0–9% (*n* = 238) as well as MDS/AML (BM or blood blasts 10–19%; *n* = 73), or AML (BM or blood blast $\ge$20%; *n* = 209) regardless of their allelic status. In the absence of comprehensive CNV analysis, CK is considered a multi-hit equivalent. Collectively, 479 (79.4%) MDS and AML with *TP53*^mut^ were classified as “*TP53*-mutated MN”. One-hundred twenty-three (20.4%) cases with *TP53*^mut^ VAF < 10% (*n* = 83) or single-hit MDS with blast 0–9% (*n* = 40) were classified along with other myeloid neoplasms (Fig. 1A–C and Supplementary Fig. 3A, B).

### *TP53*^mut^ MDS and AML cases classified differently by WHO-5 and ICC

**Fig. 2:**
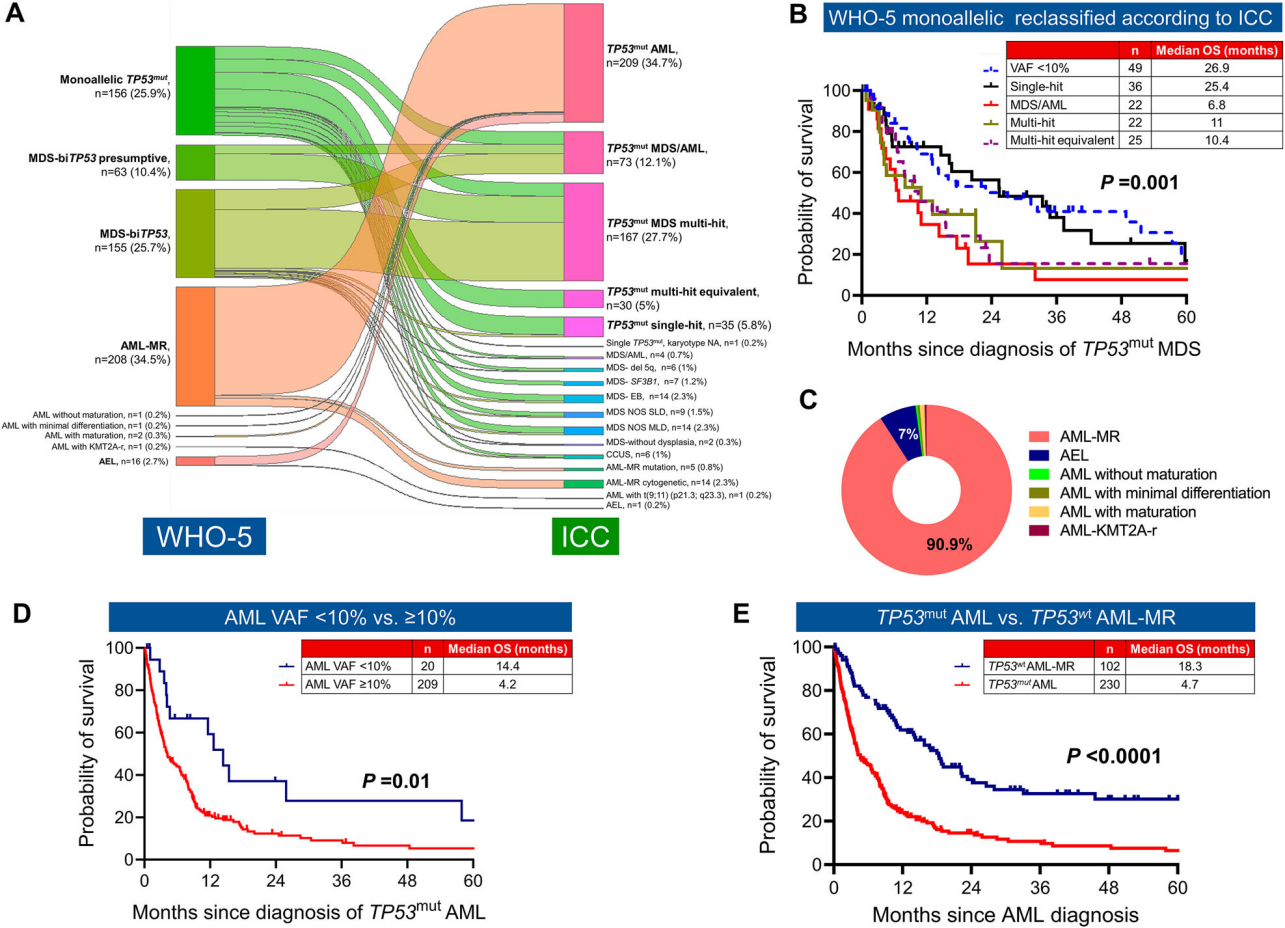
Concordance and divergence in the WHO-5 and ICC classification of *TP53*-mutated (*TP53*^mut^) myeloid neoplasm (MN). **A** Sankey plot depicting the divergences between the two classifications of *TP53*^mut^ MN. **B** heterogeneity in the overall survival (OS) of WHO-5 classified monoallelic *TP53* when reclassified using ICC criteria. **C** According to WHO-5, 90.9% of *TP53*^mut^ acute myeloid leukemia (AML) were classified as AML-myelodysplasia related (MR), followed by acute erythroid leukemia (AEL, 7%). **D** OS of AML with *TP53*^mut^ with variant allele frequency (VAF) ≥ 10% was significantly poorer compared to AML *TP53*^mut^ with VAF < 10%; and **E** OS of *TP53*^mut^ AML was significantly worse than *TP53* wild type *(TP53*^wt^*)* AML-MR.

We next focused on *TP53*^mut^ MDS and AML, that are classified differently by the two classifications. *TP53*^mut^ AML with VAF $\ge$10% (*n* = 209) were included in the ICC but not in WHO-5 as a distinct entity. Furthermore, 188 (50.4%) of 373 *TP53*^mut^ MDS cases were classified differently by ICC and WHO-5. Collectively, 407 (67.5%) cases were classified discrepantly between the two classifications (Fig. 2A).

Monoallelic MDS are excluded from the WHO-5 category of *TP53*^mut^ MDS, as their survival is considered comparable to *TP53*^wt^ MN. We analyzed WHO-5 defined 155 monoallelic *TP53*^mut^ using the ICC criteria and observed a significant spread: 49 (31.6%) had *TP53*^mut^ VAF < 10% and did not meet the ICC criteria for inclusion as *TP53*^mut^ MN; whereas 36 (23.2%), 22 (14.2%), 25 (16.1%), and 22 (14.2%) cases were classified as single-hit, multi-hit, multi-hit equivalent, and MDS/AML, respectively (Fig. 2A, B). Cytogenetic data were not available in one patient, limiting their accurate placement.

Importantly, there were significant differences in survival between these groups (*P* = 0.001, Fig. 2B), demonstrating heterogeneity in cases classified as monoallelic *TP53* loss as defined by WHO-5. Implementing the WHO-5 definition of monoallelic *TP53*^mut^ MDS would underestimate the poor prognosis of 69 (52.1%) of the 155 cases (11.4% of the entire cohort). Similarly, median OS of WHO-5 classified biallelic/presumptive biallelic MDS was significantly different when reclassified according to ICC criteria (Supplementary Fig. 4A).

### Factors driving differences in the WHO-5 and ICC of *TP53*^mut^ MDS and AML

As the discordance between the two classifications was driven by differences in the prognostic significance of: (i) *TP53*^mut^ AML; (ii) blood and bone marrow blast cut-off in MDS; (iii) 17p.13.1 deletion detected by cytogenetics; (iv) CK detected on metaphase cytogenetics; (v) *TP53*^mut^ VAF cut-off, we analyzed survival outcomes of each of these groups with an aim to provide clarity.

#### Prognostic significance of TP53^mut^ AML

AML contributed to 38.0% (*n* = 229) of the cohort. Considering the extremely poor prognosis, ICC includes *TP53*^mut^ AML with VAF $\ge$10% (*n* = 209), as a distinct entity in *TP53*^mut^ MN, regardless of allelic status; whereas *TP53*^mut^ AML with VAF < 10% (*n* = 20) were excluded from the ICC (Fig. 2C). Within our cohort, the median OS of *TP53*^mut^ AML VAF ≥ 10% was 4.2 months compared to 14.4 months for AML with *TP53*^mut^ VAF < 10% (Fig. 2D).

On the other hand, WHO-5 classifies *TP53*^mut^ AML with other AML categories and suggests further evidence is required for determining that bi*TP53* status is per se as ‘AML-defining’. Hence, applying the WHO-5 criteria, 209 (90.9%) of *TP53*^mut^ AML were classified as AML-myelodysplasia related (AML-MR), 16 (7%) as acute erythroid leukemia (AEL), and 2 (0.9%) as AML with maturation. Additionally, three cases were classified as AML without maturation (*n* = 1, 0.4%), AML with minimal differentiation (*n* = 1, 0.4%), and AML with *KMT2A*-rearrangement (*n* = 1, 0.4%) (Fig. 2A, C).

As the vast majority of *TP53*^mut^ AML would be re-classified as AML-MR according to WHO-5, we compared the survival of *TP53*^mut^ AML with *TP53*^wt^ AML-MR. The median survival of *TP53*^mut^ AML was significantly poorer compared to *TP53*^wt^ AML-MR (4.7 *vs*. 18.3 months; *P* < 0.0001) (Fig. 2E). Collectively, these results indicate that *TP53*^mut^ AML is a genetically defined subentity with extremely poor survival.

#### Prognostic significance of the blast percentage cutoffs in MDS

Another driver of divergence between the two classifications is the bone marrow and/or PB blasts cut-off. The underlying assumption by the WHO-5 is that the prognosis of bi*TP53*^mut^ MDS is homogenously poor irrespective of blast percentages between 0 and 19%. Conversely, the survival of monoallelic *TP53*^mut^ MDS, regardless of the blast category, was considered comparable to *TP53*^wt^ MDS and therefore, excluded from MDS with bi*TP53*^mut^. In contrast, the ICC acknowledges the importance of the allelic status in the context of specific blast cut-offs. For example, it mandates multi-hit status for MDS with blast 0–9%, but not for MDS/AML and AML, for inclusion in *TP53*^mut^ MN.

**Fig. 3:**
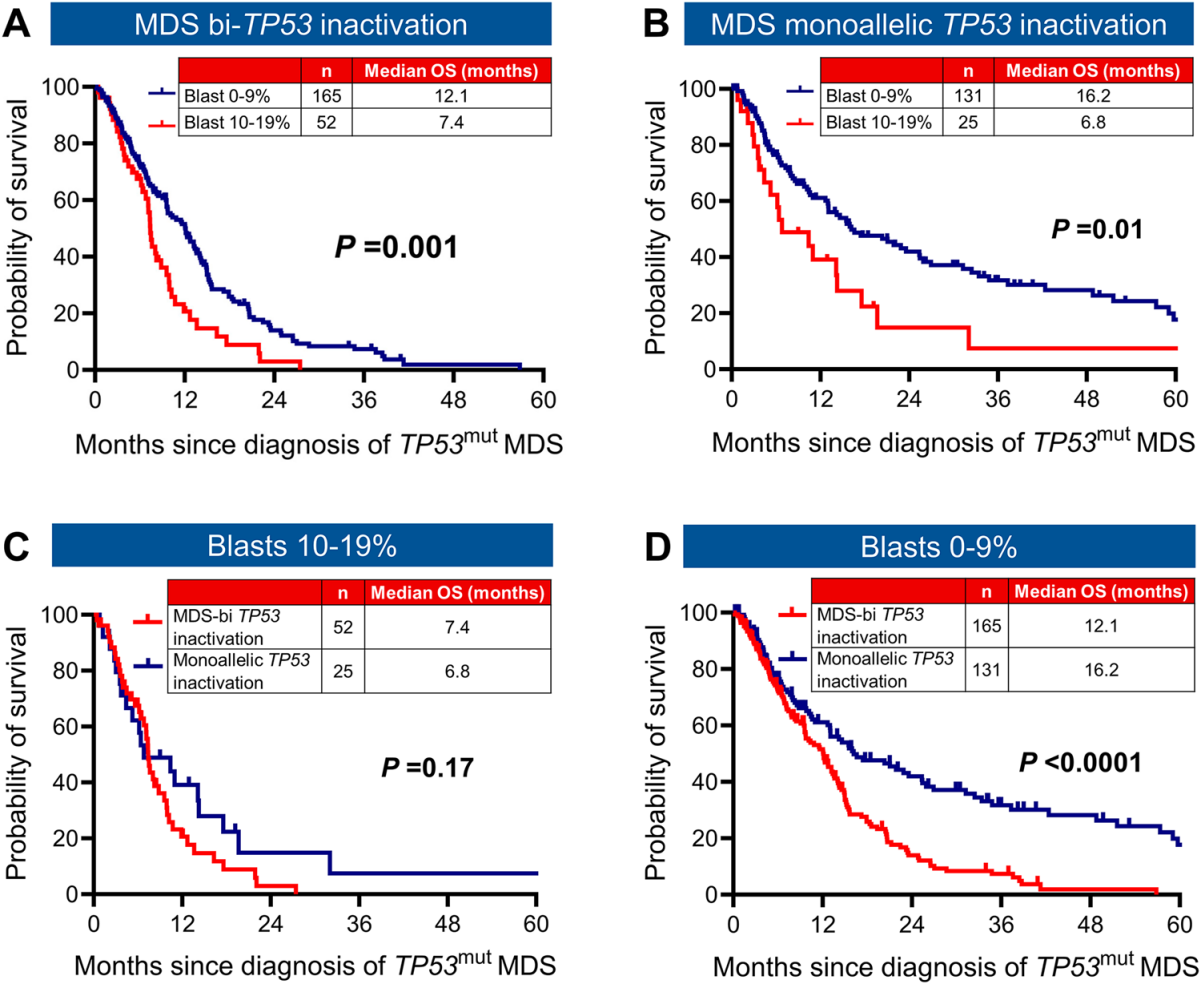
Interaction between blast cut-off and WHO-5 allelic status of *TP53*-mutated myelodysplastic syndrome (MDS). **A** Within the WHO-5 biallelic/presumptive biallelic inactivation group, the median overall survival (OS) of MDS with 10–19% blasts was significantly poorer compared to MDS 0–9% blasts. **B** Within the WHO-5 monoallelic group, the median OS of MDS 10–19% blasts was significantly poorer compared to MDS 0–9% blasts. **C** In the MDS 10–19% blast group, the median OS of biallelic/presumptive biallelic inactivation was comparable to that of monoallelic mutations. **D** While in the MDS 0–9% blast group, the median OS of biallelic/presumptive biallelic inactivation was significantly poorer compared to monoallelic mutations.

We evaluated whether the blast cut-off retains significance when the WHO-5 criteria of the allelic status were used. There was no significant OS difference between biallelic and presumptive biallelic *TP53*^mut^, hence the subgroups were combined during further analysis (Supplementary Fig. 4B). Median OS of biallelic and presumptive biallelic *TP53*-inactivation was significantly shorter for BM or blood blast 10–19% compared to 0–9% (7.4 *vs*. 12.1 months, *P* = 0.001) (Fig. 3A). Similarly, the median OS of monoallelic *TP53*^mut^ was significantly poor in cases with BM or blood blast 10–19% compared to 0–9% (6.8 *vs*. 16.2 months, *P* = 0.01) (Fig. 3B), while median OS of monoallelic and biallelic inactivation was comparable in cases with blood or bone marrow blast 10–19% (6.8 *vs*. 7.4 months; *P* = 0.17) (Fig. 3C). However, we did observe significant survival difference between biallelic inactivation and monoallelic mutations in cases with blast 0–9% (12.1 *vs*. 16.2 months; *P* < 0.0001) (Fig. 3D). Collectively, the results provide compelling evidence that blast percentage remains an important predictor of poor outcome in addition to allelic status.

**Fig. 4:**
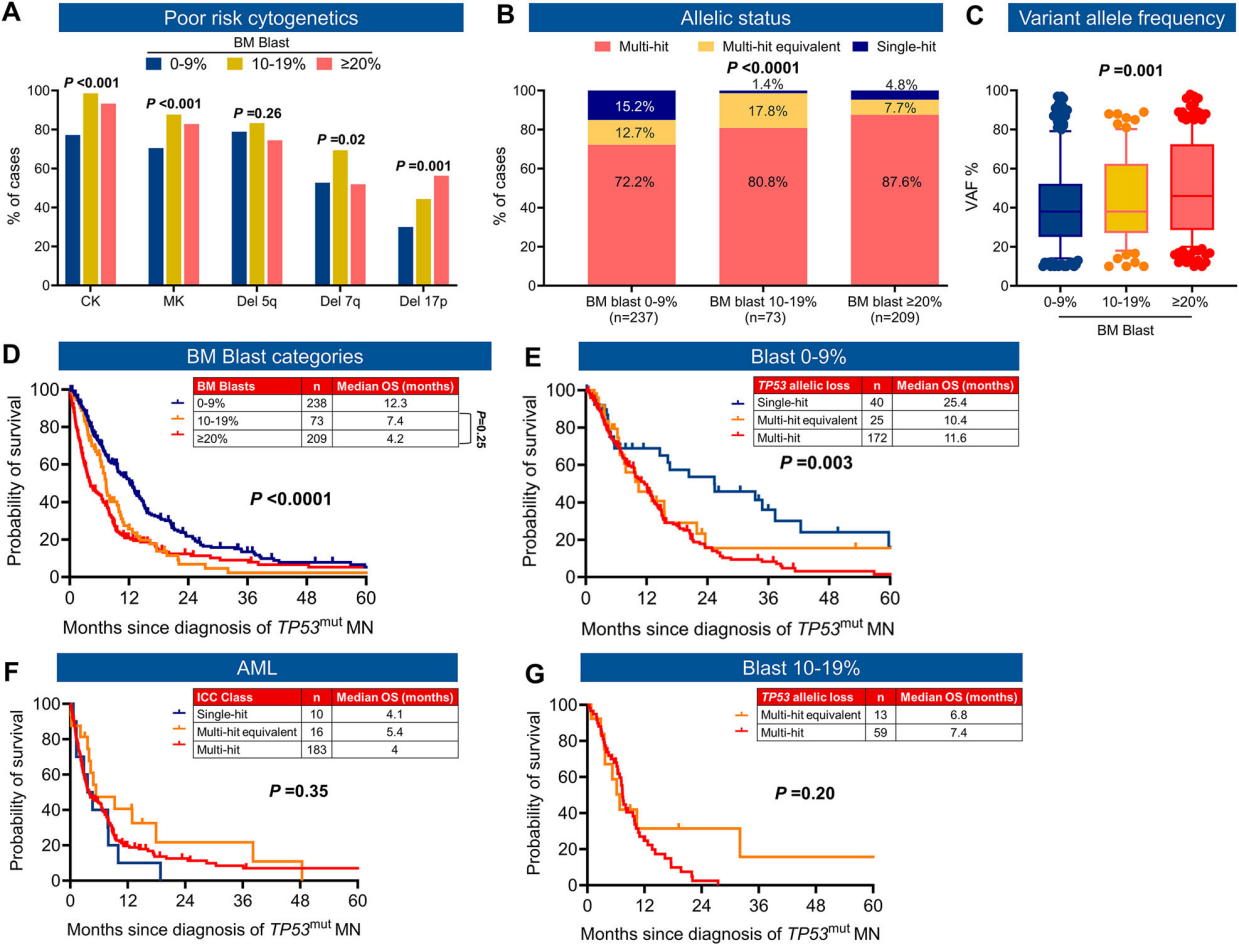
Interactions between blast percentage, *TP53* mutation (*TP53*^mut^) variant allele frequency (VAF), and allelic status based on the ICC criteria and survival of *TP53*^mut^ myelodysplastic syndrome (MDS) and acute myeloid leukemia (AML). **A** Adverse risk cytogenetic features. **B***TP53*^mut^ multi-hit and multi-hit equivalent. **C** Higher *TP53*^mut^ VAF were more prevalent in *TP53*^mut^ MDS 10–19% blasts and AML compared to MDS 0–9% blasts. **D** overall survival (OS) of *TP53*^mut^ MDS 10–19% blast and AML was significantly poorer compared to MDS 0–9% blasts. Overall survival of patients with a marrow/blood blast count of **E** 0–9%, **F** ≥20%, and **G** 10–19% according to allelic status.

We next characterized the cytogenetic profile and outcome of *TP53*^mut^ MN using ICC criteria. *TP53*^mut^ with VAF ≥ 10% (n = 520) cases included 238 (39.5%) of MDS (BM blasts 0–9%), 73 MDS/AML (12.1%), and 209 AML (34.7%). Furthermore, the impact of blast percentage is also reflected in the corroborating genomic aberrations seen with the various subgroups, along with survival outcomes. For example, poor-risk cytogenetic features such as CK, MK, 17p del, and multi-hit status were enriched in MDS/AML and AML compared to MDS (BM blasts 0–9%) (Fig. 4A, B). Similarly, *TP53*^mut^ VAF was higher MDS/AML and AML compared to MDS (Fig. 4C). The median OS of *TP53*^mut^ MDS/AML and AML was significantly poorer compared to *TP53*^mut^ MDS (BM blasts 0–9%) (4.2 *vs*. 7.4 *vs*. 12.3 months; *P* < 0.0001) (Fig. 4D). Consistent with prior reports, the median OS of multi-hit MDS (BM blasts 0–9%) was significantly poorer compared to single hit (11.6 *vs*. 25.4 months, *P* = 0.007, Fig. 4E), whilst the survival of *TP53*^mut^ AML was poor regardless of allelic status (Fig. 4F). Furthermore, across the blast percentage categories, survival was comparable between multi-hit equivalent and those with multi-hit status (Fig. 4E–G). Collectively, multi-hit or multi-hit equivalent MN had poor survival in cases with *TP53*^mut^ VAF ≥ 10%.

#### Verification of 17p13.1 loss detected on cytogenetics by CNV analysis

In cases with a single *TP53*^mut^ with VAF < 50%, WHO-5 mandates confirmation of 17p13.1 deletion by an additional CNV analysis method (e.g., FISH, SNP, or NGS), and mere detection of 17p13.1 deletion on karyotype is not sufficient to demonstrate inactivation of the other allele [15]. However, in the absence of confirmatory testing, no explicit guidelines surrounding the further classification of these cases have been provided. Conversely, the ICC does not mandate such confirmation, and both classifications do not elaborate on the need for CNV analysis in the context of a single *TP53*^mut^ with VAF < 50% without 17p13.1 deletion on karyotype.

**Fig. 5:**
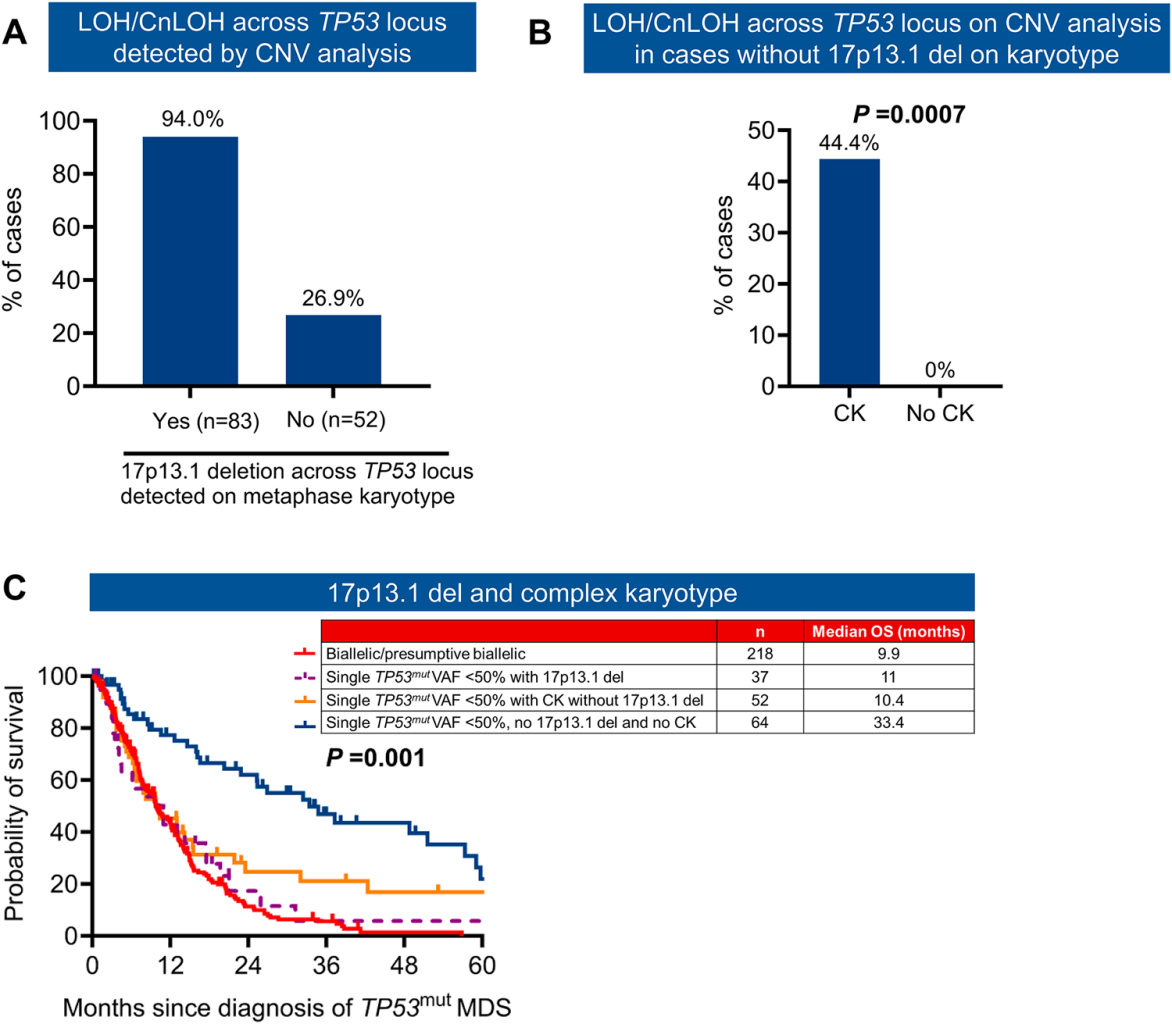
Validation and prognostic significance of 17p13.1 deletion and complex karyotype (CK) in *TP53*-mutated (*TP53*^mut^) myeloid neoplasm (MN). **A** Copy number variation (CNV) analysis confirmed 17p13.1 deletion in 94% of cases where it was observed on metaphase cytogenetics. Additionally, CNV analysis identified loss of heterozygosity (LOH) or copy-neutral LOH (CnLOH) across the *TP53* locus in 26.9% of cases without 17p13.1 deletion on metaphase cytogenetics. **B** In cases without 17p13.1 deletion detected on karyotype, LOH/CnLOH was significantly enriched in the presence of CK compared to those without CK. **C** Median overall survival (OS) of patients with a single *TP53*^mut^ with variant allele frequency (VAF) < 50% with either 17p13.1 deletion or CK was comparable to cases with biallelic/presumptive biallelic *TP53* inactivation.

Metaphase karyotype was available in 600 (99.5%) and *TP53* CNV data were available in 138 (22.9%) cases. Conventional karyotype detected loss at the *TP53* locus in 240 (40.2%) cases, and 83 (34.6%) of these had CNV analysis, which confirmed LOH in 78 (94%) of the evaluable cases. Furthermore, CNV analysis detected LOH or copy neutral LOH (cnLOH) in 14 (26.9%) of 52 evaluable cases without 17p deletion on karyotype (Fig. 5A). Importantly, in cases without 17p13.1 deletion on karyotype, striking enrichment of LOH/cnLOH (by CNV analysis) was detected in cases with CK compared to cases without CK (44.4% *vs*. 0%; *P* = 0.0007) (Fig. 5B). At the same time, all 14 cases with LOH/cnLOH across *TP53* locus on CNV, without 17p del on karyotype, had CK.

The sensitivity, specificity, positive and negative predictive value of metaphase cytogenetics to detect loss across the *TP53* locus were 84.8% (95% CI 75.8–91.4%), 88.4% (74.9–96.1%), 94.0%, and 73.1%, respectively. Collectively, these results suggest that validation of LOH across the *TP53* locus by CNV analysis may not be required in the majority of cases with 17p13.1 deletion detected on metaphase karyotype, except complex rearrangements including derivative chromosomes. In cases without 17p del on karyotype, CK can be a reasonable surrogate marker, as none of the cases without CK and 44.4% cases with CK had LOH/CnLOH across the *TP53* locus.

Next, we assessed the number of biallelic inactivations that would be underestimated in our MDS cohort in the absence of comprehensive CNV analysis. Of the 374 MDS cases, 218 (58.2%) cases had $\ge$2 *TP53*^mut^ with each VAF $\ge$2% (*n* = 115), single *TP53*^mut^ plus LOH or cnLOH across *TP53* locus (*n* = 40) or one mutation with VAF $\ge$50% (*n* = 63) and were hence able to be classified as biallelic or presumptive biallelic inactivation, respectively (Supplementary Fig. 2). The remaining 178 (47.6%) MDS had single *TP53*^mut^ with VAF < 50%. Fifty-nine cases had 17p loss on metaphase karyotype, and CNV method confirmed LOH across the *TP53* locus in 91% (*n* = 20) of 22 evaluable cases. Of the 116 (31.1%) cases with single *TP53*^mut^ VAF < 50% without 17p13.1 loss on metaphase karyotype, CNV information was available in 20 cases, and 3 (15%) cases had LOH (*n* = 1) or cnLOH (*n* = 2). Importantly, LOH/cnLOH detected by CNV analysis was enriched in cases with single *TP53*^mut^ VAF < 50% with CK without 17p13.1 loss on karyotype compared to their counterparts without CK (33.3% *vs*. 0%; *P* = 0.07). In summary, in the absence of comprehensive CNV data, biallelic inactivation would have been missed in 14 cases with single *TP53*^mut^ VAF < 50% with CK but without 17p loss, which accounts for 4.0% of the total 374 MDS cases. However, the absence of this information is less likely to underestimate the poor prognosis of these cases, as shown below, as the median OS of single *TP53*^mut^ VAF < 50% with CK is poor and comparable to biallelic inactivation.

#### Prognostic significance of 17p loss and CK detected on metaphase cytogenetics

Of the 178 MDS with *TP53*^mut^ VAF < 50%, 37 (20.8%) had 17p loss on karyotype, and the median OS of these cases was comparable to biallelic or presumed biallelic *TP53* loss (11.0 *vs*. 9.9 months; *P* = 0.63) but significantly poorer than those with monoallelic *TP53*^mut^ VAF < 50% without 17p loss and/or CK on metaphase cytogenetics (9.9 *vs*. 33.4 months; *P* < 0.0001) (Fig. 5C). Furthermore, 52 (29.2%) cases had CK without 17p loss on karyotype analysis, while 64 (40.0%) cases did not exhibit either abnormality. The median OS of single *TP53*^mut^ VAF < 50% with CK without 17p loss (*n* = 52) was comparable to those with 17p13.1 deletion on karyotype (10.4 *vs*. 11.0 months; *P* = 0.39) but significantly poorer than those cases with monoallelic *TP53*^mut^ without 17p loss or CK (10.4 *vs*. 33.4 months; *P* < 0.0001) (Fig. 5C). Collectively, these results indicate that in MDS cases with single *TP53*^mut^ VAF < 50%, the presence of CK can be considered a practical surrogate for biallelic *TP53* inactivation.

#### Differences in classification based on TP53^mut^ VAF cut-off

The ICC mandates the inclusion of *TP53*^mut^ with VAF $\ge$10% into the category of *TP53*^mut^ MN, whereas the WHO-5 does not have a similar threshold. Within our cohort, 83 (13.8%) of 603 *TP53*^mut^ MDS and AML had *TP53*^mut^ VAF < 10% and were not classified as *TP53*^mut^ MN by ICC (Fig. 1A). These 83 cases included AML (*n* = 21), MDS/AML (*n* = 4) and MDS with 0–9% blasts (*n* = 58), which are currently classified by ICC as AML-MR (*n* = 20), MDS-EB (*n* = 14), MDS not otherwise specified (NOS) multilineage dysplasia (MLD, *n* = 14), MDS NOS single lineage dysplasia (SLD, *n* = 9), MDS del 5q (*n* = 6), MDS *SF3B1* (*n* = 7) and clonal cytopenia of undetermined significance (CCUS) (*n* = 6).

**Fig. 6:**
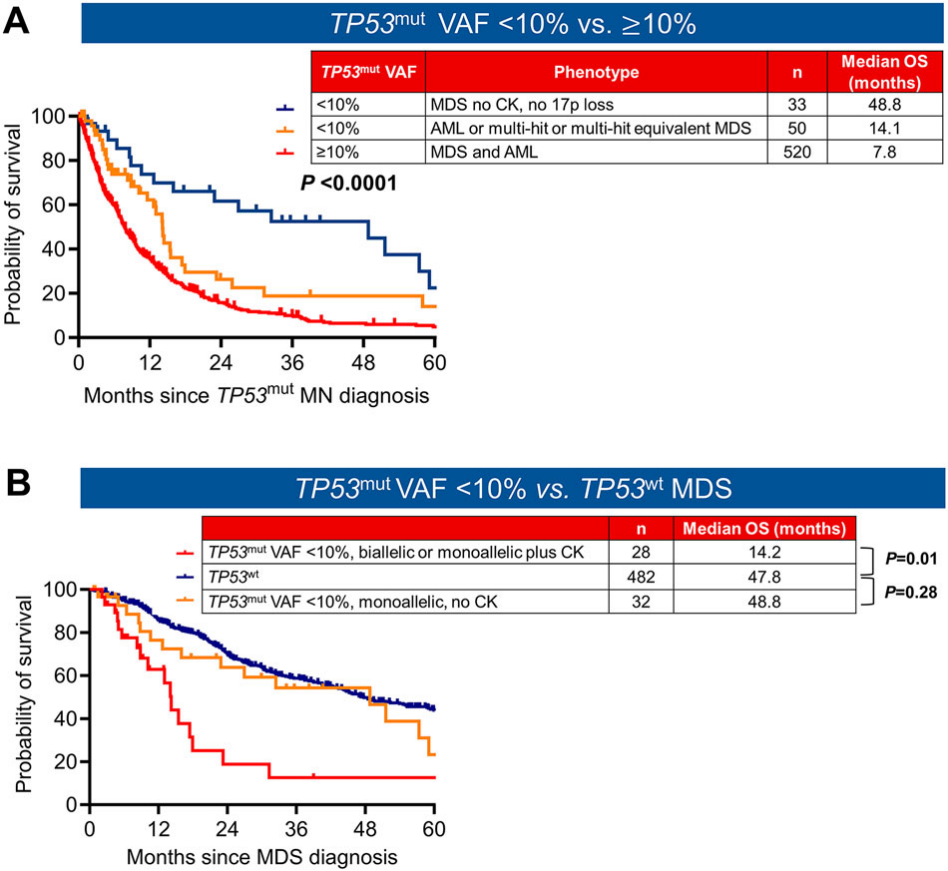
The median OS of biallelic or single *TP53*^mut^ VAF < 10% plus CK was significantly shorter compared to that of single *TP53*^mut^ VAF < 10% without CK and was comparable to *TP53*^mut^ VAF ≥ 10%. **A** The median OS of AML or multi-hit/multi-hit equivalent MDS was significantly worse compared to MDS cases with a single *TP53*^mut^ VAF < 10% without 17p loss or CK, and was comparable to *TP53*^mut^ VAF ≥ 10%. **B** In a *TP53*^mut^ VAF < 10% MDS, the median OS of biallelic or monoallelic with CK was significantly poor. While the median OS of monoallelic *TP53*^mut^ VAF < 10% without CK was comparable to *TP53* wild type (*TP53*^wt^) MDS.

The median OS of *TP53*^mut^ AML, MDS with $\ge$2 *TP53*^mut^ or single *TP53*^mut^ plus 17p13.1 deletion and/or CK (*n* = 50) was significantly shorter compared to that of single *TP53*^mut^ VAF < 10% without CK and 17p loss (*n* = 33) and was comparable to *TP53*^mut^ VAF ≥ 10% (14.1 *vs*. 48.8 *vs*. 7.8 months, *P* < 0.0001) (Fig. 6A), whereas the median OS of monoallelic *TP53*^mut^ VAF < 10% without CK was comparable to *TP53*^wt^ MDS (Fig. 6B).

A recent study suggested that a *TP53*^mut^ VAF $\ge$20% is associated with poor outcome and may be considered as presumptive biallelic [17]. In line with the literature, the median OS of *TP53*^mut^ VAF $\ge$20% was significantly poorer compared to *TP53*^mut^ VAF < 20% (7.6 *vs*. 14.4 months; *P* < 0.0001) (Supplementary Fig. 5A). However, VAF $\ge$20% should not be equated with biallelic equivalent due to the complex interaction of VAF, allelic status, blast percentage, and CK.

For cases with biallelic and monoallelic *TP53*^mut^ plus CK, the median OS was poor regardless of whether the VAF was $\ge$20% or <20% (9.6 *vs*. 14.1 months; *P* = 0.01), although it was shorter in the VAF $\ge$20% group. In contrast, for monoallelic *TP53*^mut^ without CK, the median OS was significantly longer regardless of VAF $\ge$20% *vs*. < 20% (33.4 *vs*. 37.3 months; *P* = 0.64) (Supplementary Fig. 5B). This highlights that biallelic status remains a key driver of poor prognosis, irrespective of VAF.

The poor prognosis of VAF $\ge$20% is primarily due to the enrichment of MDS-EB2, AML, CK, biallelic, and monoallelic inactivation with CK (Supplementary Fig. 5C–E). However, monoallelic *TP53*^mut^ without CK and VAF $\ge$20% are associated with longer survival. Therefore, considering *TP53*^mut^ VAF $\ge$20% as equivalent to biallelic inactivation could overestimate the poor prognosis of monoallelic mutation without CK.

## Discussion

The classification of *TP53*^mut^ MN is aimed at improving diagnostic and prognostic accuracy, guiding therapeutic decision-making, advancing research, and facilitating clinical trial design and enrollment. However, discrepancies between classifications can delay diagnosis, lead to divergent treatment approaches, and hinder drug development.

We systematically investigated discordances between the two classifications and provided novel evidence to refine subsequent iterations. The urgency of such an effort is evident given that 64% of *TP53*^mut^ MN cases were not classified as such by WHO-5, compared to 20% by ICC. Additionally, 67.5% of *TP53*^mut^ MN cases, including 50% of MDS, were classified differently by the two systems.

Our analysis highlights that the clinical significance of biallelic *TP53* inactivation is highly context-dependent and influenced by blast percentage. The discrepancy between WHO-5 and ICC emphasizes this issue: the former considers MDS with 0–19% BM/PB blasts as a homogenous group, mandating the demonstration of biallelic *TP53* inactivation regardless of blast percentage. In contrast, ICC mandates the evidence of multi-hit *TP53* loss for cases with 0–9% blasts, but single *TP53*^mut^ VAF ≥ 10% is sufficient to designate MDS/AML and AML as *TP53*^mut^ MN. First, we show that the survival of the 10–19% blast subset was poor regardless of allelic status; whereas the survival of biallelic *TP53*^mut^ MDS with 0–9% blasts was significantly poorer compared to the monoallelic cases. These findings follow recent reports from our [18] and other groups [3] that the 0–9% blast category is not homogenous and MDS-low blast (MDS-LB, <2% blood blast and <5% BM blast) exhibit distinct biological and clinical characteristics compared to MDS-excess blast 1 (MDS-EB1, 2–4% blood blast and 5–9% BM blast) [18] and the significance of biallelic inactivation was restricted to MDS-LB but not for MDS-EB1 or MDS-EB2. Furthermore, MDS-EB1 and -EB2 with VAF ≥ 10% had comparable survival outcomes, regardless of allelic status. Similarly, Stengel et al. also observed that MDS with <5% blasts differed biologically and prognostically from cases with ≥5% to <10% blasts, ≥10% to <20% blasts, and AML. Specifically, *TP53* single-hit cases were more common in the blast <5% group, while *TP53* double-hit cases dominated the >5% blast groups, with frequencies of 67%, 91% and 71% in the ≥5% to 9%, and ≥10% to 19%, and AML categories, respectively [3]. In contrast to their prior report, Bernard et al. [2] recently reported that the *TP53*^mut^ cases can be further stratified by blast categories with a significant OS difference. In their cohort, the median OS was 1.4, 0.8, and 0.7 years in cases with 0–5%, 5–10%, and 10–20% bone marrow blasts, respectively [19]. However, the International Consortium for MDS and the TITAN study proposed a single group of biallelic *TP53*^mut^ regardless of blast categories (bone marrow blast <20%) [20, 21]. In summary, the prognostic implication of blast percentage remains an area of active research.

In the current form, the WHO-5 may underestimate the poor prognosis of monoallelic *TP53*^mut^ cases with 10–19% blasts. Consequently, such patients may be denied potentially curative therapies, such as allogeneic stem cell transplant, as they are misclassified as having median OS comparable to *TP53*^wt^ cases. It further follows that clinical trials evaluating novel therapeutics should continue to stratify patients based on blast percentage categories rather than handling them as a single group. For instance, achieving a median OS of 12–14 months, which may represent a substantial improvement for patients with 10–19% blasts, with current predicted survival of ~7.5 months, but not for patients with 0–9% blasts with predicted survival of ~12 months.

*TP53*^mut^ AML is frequently associated with adverse cytogenetics and has extremely poor survival [13, 22, 23] and is recognized as a distinct subcategory in the ICC [16]. On the other hand, WHO-5 [24] classifies *TP53*^mut^ AML within ‘other AML categories’, stating that further evidence is required to confirm bi*TP53* status as an ‘AML-defining abnormality’. Consequently, WHO-5 would instead classify >90% of *TP53*^mut^ AML as AML-MR. We note that the survival of *TP53*^mut^ AML was significantly poorer compared to *TP53*^wt^ AML-MR. A similar observation was recently reported by Hart et al.. The clinical implication of these findings is that *TP53*^mut^ AML may, inadvertently, be excluded from clinical trials of novel therapeutics if the WHO-5 criteria are followed. Secondly, when combined as a single entity, this misclassification could lead to prognostic overestimation and underestimation of *TP53*^mut^ AML and *TP53*^wt^ AML-MR, respectively.

In cases with one *TP53*^mut^ VAF < 50%, demonstrating loss of the other copy of *TP53* is critical to establish multi-hit loss. While the WHO-5 classification mandates verification of 17p13.1 deletion detected by metaphase cytogenetics using a confirmatory CNV analysis [15], the ICC does not [16]. Current practice reflects substantial limitations, with only ~20% of patients at our tertiary cytogenetics laboratories having undergone the confirmatory CNV analysis. We found that the confirmatory CNV analysis verified the LOH across the *TP53* locus in 94% of evaluable cases with 17p13.1 deletion detected by metaphase cytogenetics. Notably, the survival of cases with single *TP53*^mut^ VAF < 50% and concurrent 17p loss on karyotype was comparable to that of cases with biallelic *TP53* inactivation. Additionally, CNV analysis identified LOH/cnLOH in 26.9% of evaluable cases without 17p deletion on karyotype, consistent with findings by Bernard et al. [2] ( ~ 25%). Strikingly, cases without 17p13.1 deletion on karyotype but with LOH/cnLOH on CNV coexisted with CK only. All cases with LOH/cnLOH across the *TP53* locus on CNV analysis without 17p deletion on karyotype had CK. Conversely, no LOH/cnLOH was observed in cases with single *TP53*^mut^ (VAF < 50%) without CK on karyotype. In the absence of comprehensive CNV data, biallelic inactivation would have been missed in approximately 4.0% of 374 MDS, primarily among those with single *TP53*^mut^ VAF 2–50% and CK but without 17p loss. However, the prognostic impact of this omission is expected to be limited, as the median OS of single *TP53*^mut^ VAF < 50% with CK is poor and comparable to biallelic inactivation. Our findings demonstrate that 17p13.1 deletion and/or CK detected by karyotype is associated with poor prognosis and may be considered “presumptive biallelic *TP53* loss”. Validation in larger, independent cohorts will enhance the utility in clinical practice.

Finally, the ICC mandates a *TP53*^mut^ VAF threshold of ≥10%, whereas WHO-5 does not specify a VAF cut-off. Our study provides compelling evidence that multiple *TP53*^mut^, each with VAF < 10%, as well as single *TP53*^mut^ VAF < 10% in the context of 17p loss or CK, as well as *TP53*^mut^ AML, are associated with poor survival. These findings suggest that the ICC-recommended *TP53*^mut^ VAF cut-off of ≥10% warrants reconsideration, and cases with biallelic and monoallelic with CK with VAF 2 to 10% should be included in *TP53*^mut^ MN.

The findings of our retrospective study, spanning over two decades, reflect the diagnostic workup, therapeutic sequencing, and clinical decision-making influenced by institutional guidelines of the time. This includes limited utilization of confirmatory CNV analysis. It is possible that workflow mandating confirmatory CNV analysis and/or wider utilization of whole genome sequencing may result in a higher proportion of cases being considered multi-hit [25]. Another limitation is the lack of bone marrow fibrosis, and hence, we could not evaluate the association between *TP53*^mut^ MN and marrow fibrosis. Our study demonstrates a poor outcome of *TP53*^mut^ MN with the currently available therapies. Although allogeneic SCT is associated with longer survival compared to other disease-modifying therapies, the median OS following allogeneic SCT is only ~1 year. The high relapse rate following allogenic SCT remains a pressing challenge, and our group has previously investigated the factors associated with this high relapse rate [26].

Despite these limitations, our analysis of the largest cohort of MN harboring *TP53*^mut^ to date addresses key areas of contention and provides valuable insights that may guide future revisions of the WHO and ICC classifications. For the WHO-5 revision, we propose recognizing 17p13.1 deletion or a CK identified through conventional karyotyping as indicative of biallelic inactivation or its equivalent, without requiring confirmation by CNV analysis in the vast majority of cases. Furthermore, the poor prognosis associated with monoallelic *TP53* inactivation in MDS cases with 10–19% blasts should be acknowledged, and *TP53*^mut^ AML should be included in the *TP53*^mut^ MN category. For the ICC, we propose revisiting the *TP53*^mut^ VAF threshold of ≥10%, as multi-hit, multi-hit equivalent *TP53*^mut^ MDS, and AML with VAF < 10% demonstrated survival outcomes comparable to those with VAF ≥ 10% (summarized in Table 1). This highlights the need for a more nuanced classification. Both the WHO-5 and ICC classifications should also reconsider the blast percentage cut-off and allelic status, as emerging data suggest that MDS-LB has distinct disease biology and outcomes compared to MDS with 5–9% blasts, 10–19% blasts, and AML.

## Supplementary information

Supplementary methods and results

## Data Availability

Additional methods and data can be found in the Supplementary Methods section. For original data, please contact devendra.hiwase@sa.gov.au or shah.mithun@mayo.edu.
