# Supplementary material for: Validation of the 5th edition of the World Health Organization and International Consensus Classification guidelines for TP53-mutated myeloid neoplasm in an independent international cohort
Source: Blood Cancer J. 2025 May 7;15(1):88. doi: 10.1038/s41408-025-01290-0 (PMC12059121; doi:10.1038/s41408-025-01290-0)
Supplement: Supplementary file 1 — Supplementary methods and results [file 41408_2025_1290_MOESM1_ESM.docx]

**Supporting Information for:**

**Validation of the 5^th^ edition of the World Health Organization and International Consensus Classification guidelines for *TP53*-mutated myeloid neoplasm in an independent international cohort**

**This file contains:**

**Methods**

**Figure S1 – S5**

**References**

**SUPPLEMENTARY METHODS, TABLES & FIGURES**

**Methods**

**Patient samples.** Collaboration between Mayo clinic (Rochester, USA) and South Australian MDS/AML registry (Australia) led to this International Registry of *TP53* mutated myeloid neoplasm (n=603) including MDS (n=374, 62%) and AML (n=229, 38%). Data was obtained with informed consent or appropriate consent waiver in accordance with the Declaration of Helsinki and appropriate Ethics Committee approval.

**Clinical data.** All data shared for this study were assigned unique patient identifiers and anonymized data including clinical variables such as blood counts, bone marrow blasts, conventional G-banding karyotypes, pathogenic somatic mutations, clinical outcomes including last follow-up, survival status, and the time of AML transformation.

**Cytogenetics and other analyses.** Conventional karyotype analysis was carried out as described previously (1, 2). Complex karyotype (CK) was defined as previously described. Monosomal karyotype (MK) was defined by the presence of 2 or more monosomies (excluding loss of X or Y), or 1 monosomy plus at least 1 structural chromosomal aberration (excluding AML-associated recurrent cytogenetic abnormality (1, 2).

**Fluorescence in situ hybridization (FISH).** FISH analysis was performed to verify loss of *TP53* locus in cases with 17p13.1 deletions as described previously. FISH findings were reported in accordance with the 2017 International System for Human Cytogenetic Nomenclature.

**SNP array***.* Microarray-based genomic profiling was performed on BM-MNC using Affymetrix CytoScan HD platform and Illumina CytoSNP-850K BeadArray v1.2. These arrays comprise approximately 750,000 and 850,000 SNP markers retrospectively across the whole genome. Following hybridization of PCR-amplified, enzymatically fragmented and labelled DNA to the complementary oligomers, the Cytoscan HD chip was imaged, and the resulting files were analysed using Chromosome Analysis Suite software (ChAS) and BlueFuse Multiv4.5 with genome build GRCh37 with backbone resolution ~50 Kb and targeted gene average resolution ~10Kb. CN was determined by the signal intensity of all 2.67 million markers and genotyping was assessed by signal intensities for the A and B alleles, yielding allelic values.

*Copy Number variation (CNV) detection.* CNV detection using NGS was performed using an in-house developed algorithm as previously described (3, 4).

*Copy neutral loss of heterozygosity (cnLOH) detection.* Where applicable, patient samples with no copy number abnormalities were checked for evidence of cnLOH by informative SNPs in genes either side of the *TP53* gene from available NGS data.

**Next-generation sequencing (NGS) panels.** DNA was extracted from bone marrow aspirates and sequencing was performed using a targeted NGS panel at each institution (5-7).

Briefly, at *Mayo Clinic,* libraries were prepared using the Agilent SureSelect‐XT Target Enrichment Kit (SureSelectXT, Agilent, Santa Clara, CA) and sequencing was performed on MiSeq or HiSeq platforms (Illumina, San Diego, CA) at the Mayo Clinic Clinical Genome Sequencing Laboratory.

For the South Australian cohort, libraries were prepared using NimbleGen Capture Platform (Roche NimbleGen Inc., Madison, WI, USA) and sequenced on an Illumina HiSeq2500 sequencing system (Illumina). Entire coding regions were sequenced for each gene. Only variants with a total read depth >50, supported by more than five alternate variant reads and a variant allele frequency (VAF) ≥2%, were retained for further analysis. Pathogenic and likely pathogenic variant~~s~~ calling was performed as described (8). Somatic variants were classed as oncogenic as previously described (8, 9).

Frequently mutated genes in myeloid malignancies and sequenced in both cohorts were analyzed: *ASXL1, BCOR, CBL, CEBPA, DDX41, DNMT3A, EZH2, FLT3, GATA2, IDH1, IDH2, JAK2, KIT, KRAS, MPL, NPM1, NRAS, PTPN11, RUNX1, SETBP1, SF3B1, SRSF2, TET2, TP53, U2AF1, WT1* and *ZRSR2.*

**
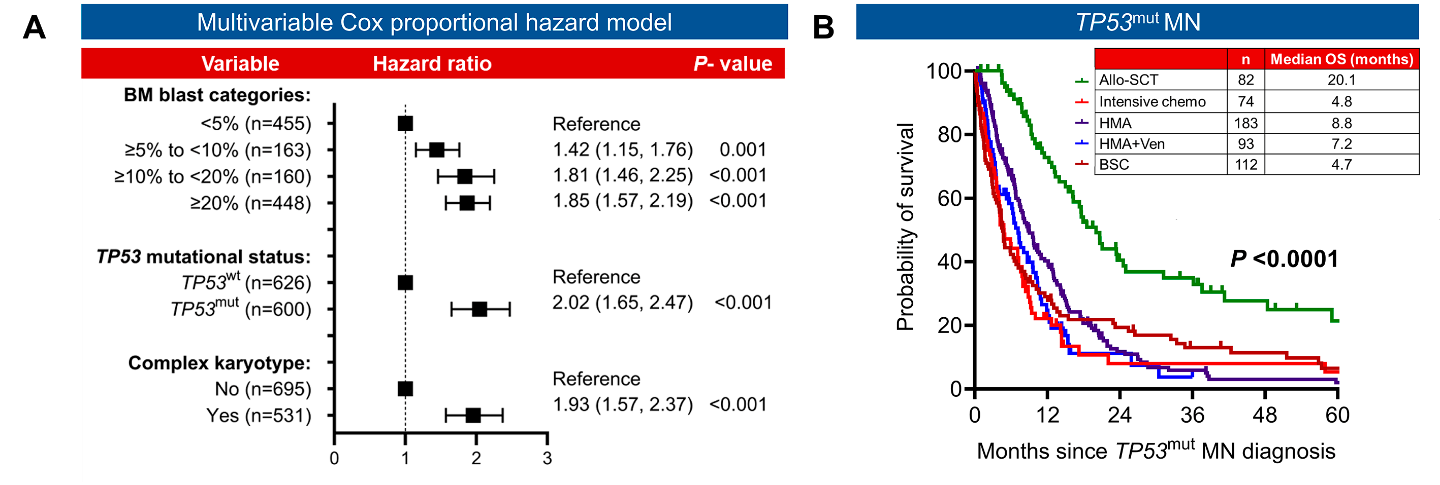
**

**Supplementary Figure 1: *TP53*^mut^ is associated with poor survival regardless of disease modifying therapies**. (A) in a multivariate Cox proportional hazard model *TP53*^mut^ is associated with poor survival independent of complex karyotype (CK) and blast percentage; (B) allogeneic SCT was associated with longer OS in *TP53*-mutated myeloid neoplasm compared to intensive chemotherapy, hypomethylating agents (HMA) with or without venetoclax (HMA + Ven) was associated with poor outcome.


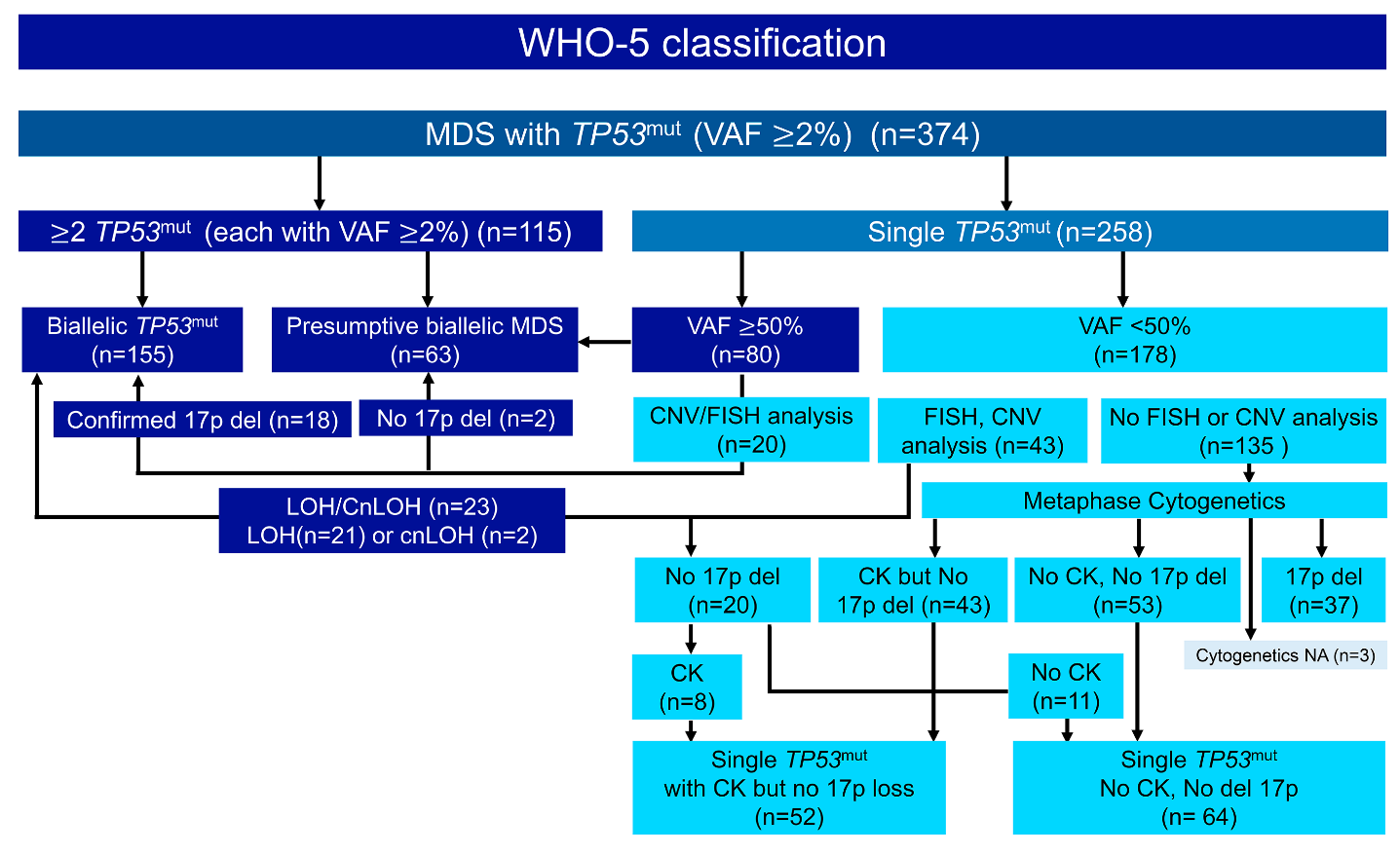


**Supplementary Figure 2**. **Consort diagram showing distribution of *TP53*^mut^ MDS according to WHO-5.** Classification of ***TP53*^mut^** MDS by utilizing WHO-5 criteria and 17p loss on karyotype.

**
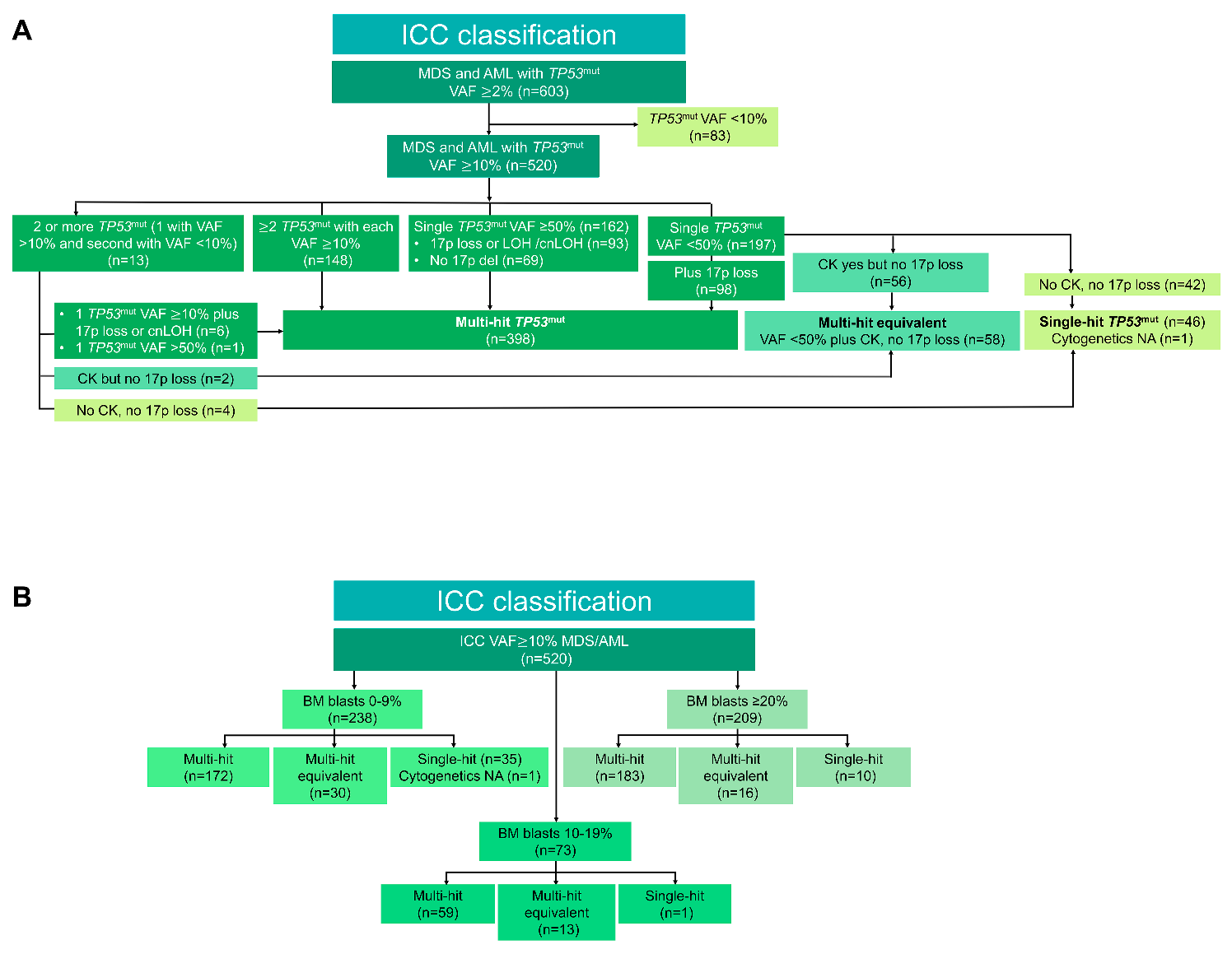
**

**Supplementary Figure 3.** **Consort diagram showing distribution *TP53*^mut^ MN according to ICC.** (A) Annotations of allelic status for *TP53*^mut^ MN by utilizing ICC criteria; (B) flowchart delineating single- *vs.* multi-hit status in *TP53*^mut^ MN according to ICC bone marrow/blood blast categories.

**
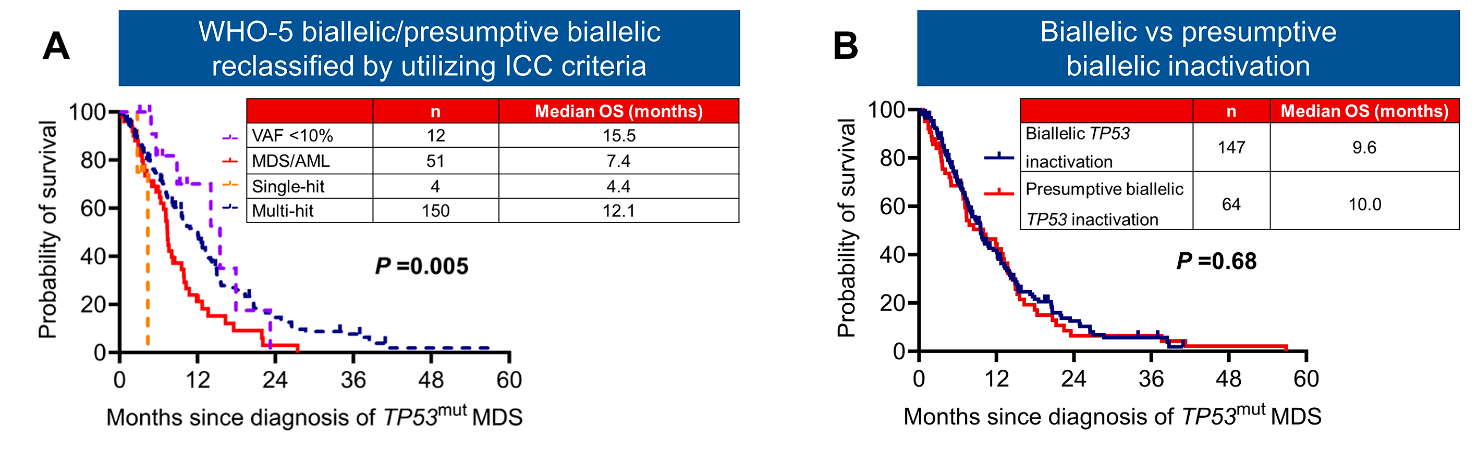
**

**Supplementary Figure 4.** **WHO-5 defined biallelic/presumptive biallelic inactivation reclassified by utilizing ICC criteria.** (A) WHO-5 defined biallelic/presumptive biallelic can be further classified by ICC criteria with significant survival difference; (B) median survival of biallelic and presumptive biallelic MDS is similar.


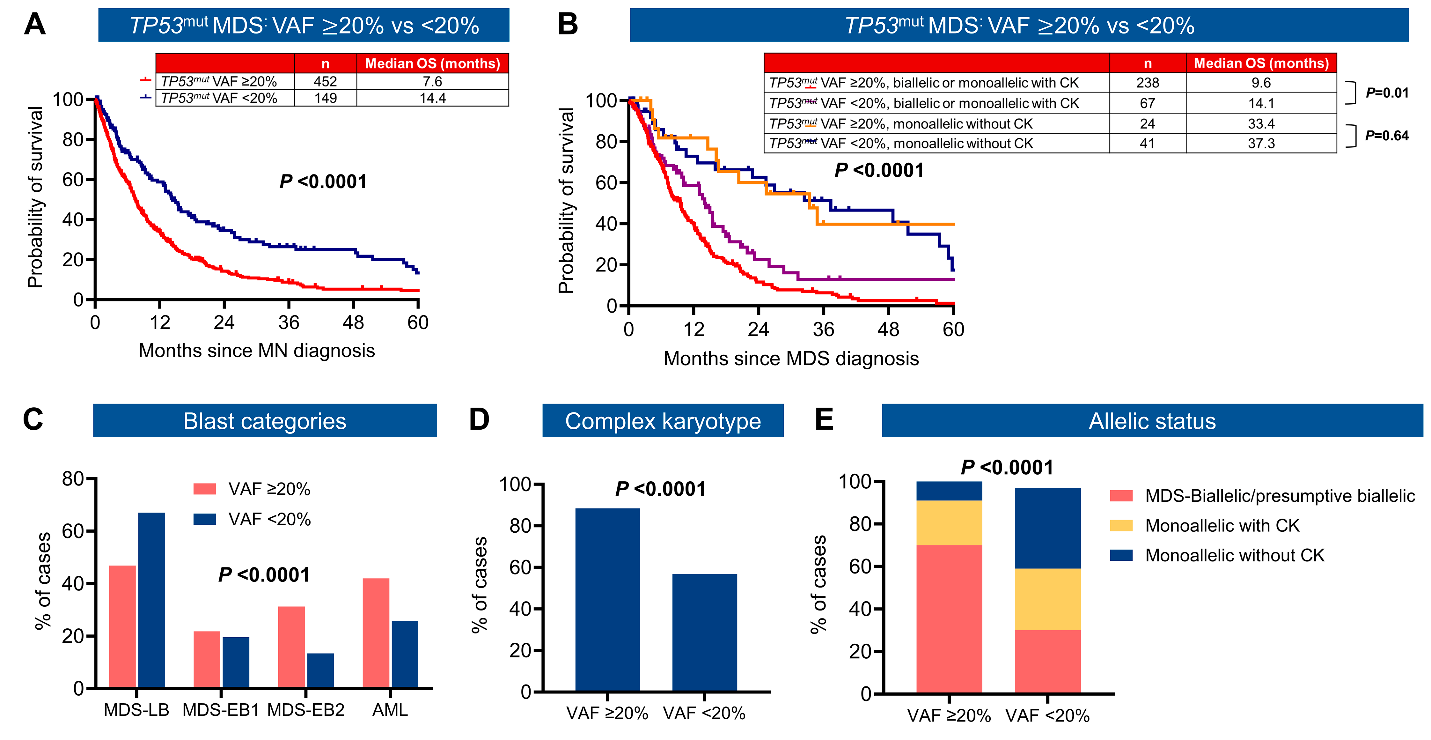


**Supplementary Figure 5**: **Poor prognosis of *TP53*^mut^ MN VAF** $\boldsymbol{\geq}$**20% is probably driven by enrichment of biallelic, CK and high blast MN.** (A) Significantly poor OS of *TP53*^mut^ MN VAF $\geq$20% compared to <20%; (B) median OS of biallelic and monoallelic plus CK was poor regardless of *TP53*^mut^ VAF $\geq$20% *vs.* <20%. In contrast, median OS monoallelic without CK was comparable and significantly longer regardless of *TP53*^mut^ VAF $\geq$20% *vs.* <20%. The poor survival is probably driven by enrichment of (C) AML and MDS-EB2, (D) CK, (E) biallelic and monoallelic plus CK in cases with *TP53*^mut^ VAF $\geq$20%, compared to VAF <20%.

**References**

1. Chun K, Hagemeijer A, Iqbal A, Slovak ML. Implementation of standardized international karyotype scoring practices is needed to provide uniform and systematic evaluation for patients with myelodysplastic syndrome using IPSS criteria: An International Working Group on MDS Cytogenetics Study. Leuk Res. 2010;34(2):160-5.

2. McGowan-Jordan J, Hastings R, Moore S. Re: International System for Human Cytogenetic or Cytogenomic Nomenclature (ISCN): Some Thoughts, by T. Liehr. Cytogenet Genome Res. 2021;161(5):225-6.

3. Branford S, Wang P, Yeung DT, Thomson D, Purins A, Wadham C, et al. Integrative genomic analysis reveals cancer-associated mutations at diagnosis of CML in patients with high-risk disease. Blood. 2018;132(9):948-61.

4. Hiwase DK, Hahn CN, Tran ENH, Chhetri R, Baranwal A, Al-Kali A, et al. TP53 mutation in therapy-related myeloid neoplasm defines a distinct molecular subtype. Blood. 2022.

5. Hiwase D, Hahn C, Tran ENH, Chhetri R, Baranwal A, Al-Kali A, et al. TP53 mutation in therapy-related myeloid neoplasm defines a distinct molecular subtype. Blood. 2023;141(9):1087-91.

6. Shah MV, Tran ENH, Shah S, Chhetri R, Baranwal A, Ladon D, et al. TP53 mutation variant allele frequency of ≥10% is associated with poor prognosis in therapy-related myeloid neoplasms. Blood Cancer J. 2023;13(1):51.

7. Singhal D, Wee LYA, Kutyna MM, Chhetri R, Geoghegan J, Schreiber AW, et al. The mutational burden of therapy-related myeloid neoplasms is similar to primary myelodysplastic syndrome but has a distinctive distribution. Leukemia. 2019;33(12):2842-53.

8. Li MM, Datto M, Duncavage EJ, Kulkarni S, Lindeman NI, Roy S, et al. Standards and Guidelines for the Interpretation and Reporting of Sequence Variants in Cancer: A Joint Consensus Recommendation of the Association for Molecular Pathology, American Society of Clinical Oncology, and College of American Pathologists. J Mol Diagn. 2017;19(1):4-23.

9. Mehta N, He R, Viswanatha DS. Internal Standardization of the Interpretation and Reporting of Sequence Variants in Hematologic Neoplasms. Mol Diagn Ther. 2021;25(4):517-26.
